# A Privacy-Preserved Variational-Autoencoder for DGA Identification in the Education Industry and Distance Learning

**DOI:** 10.1155/2022/7384803

**Published:** 2022-03-24

**Authors:** Xingxing Zheng, Xiaona Yin

**Affiliations:** Zhengzhou Preschool Education College, Zhengzhou 450000, China

## Abstract

One of the most insidious methods of bypassing security mechanisms in a modern information system is the domain generation algorithms (DGAs), which are used to disguise the identity of malware by periodically switching the domain name assigned to a command and control (C&C) server. Combating advanced techniques, such as DGAs, is an ongoing challenge that security organizations often need to work with and possibly share private data to train better and more up-to-date machine learning models. This logic raises serious concerns about data integrity, trade-related issues, and strict privacy protocols that must be adhered to. To address the concerns regarding the privacy and security of private data, we propose in this work a privacy-preserved variational-autoencoder to DGA combined with case studies from the education industry and distance learning, specifically because the recent pandemic has brought an explosive increase to remote learning. This is a system that, using the secured multi-party computation (SMPC) methodology, can successfully apply machine learning techniques, specifically the Siamese variational-autoencoder algorithm, on encrypted data and metadata. The method proposed for the first time in the literature facilitates learning specialized extraction functions of useful intermediate representations in complex deep learning architectures, producing improved training stability, high generalization performance, and remarkable categorization accuracy.

## 1. Introduction

The pandemic has had an impact on how people learn and on all stakeholders involved. The functioning of higher education institutions has been harmed. Many institutions are currently unable to perform examinations because of the pandemic, and the face-to-face teaching and learning process has been harmed. The current scenario is projected to last for some time, so it is critical to improve the learning process by establishing strategies with respect to online learning, preserving social distance, and, of course, the privacy of the data exchanged online by the various stakeholders [1, 2]. Researchers have been focusing on the growth of distance education in recent years, but because of the COVID-19 pandemic, distance learning has become a critical task for the education system and the privacy related issues.

For several years, botnet technology has been the mainstay of orchestrating and supporting a wide variety of cyber-attacks, such as DDoS attacks, and phishing. Malicious botnet masters use DGAs extensively to make it possible for the C&C server to communicate with their bots in such a way as to bypass the known malware detection mechanisms. Typically, a DGA algorithm, using a seed known only to devices participating in the botnet, periodically generates, at random times, a pseudo-random set of domain names, which act as candidates for assignment to the C&C server [3]. As a result, traditional static botnet handling techniques are becoming ineffective as the pace at which C&C changes names is unable to detect and terminate communication in time. Examples of such static techniques include blacklisting the static domain name of a C&C server as soon as it is detected, reverse-engineering the malware of an infected device to reconstruct the DGA used roughly and studying how the malware generates names. Ideally, however, malicious names should be detected in real-time, with the predictions being performed at the level of individual names, to avoid establishing bots' communication with the C&C server [4].

The field of machine learning has dramatically attracted the interest of cyber security researchers to address this problem [5]. In previous approaches based on machine learning methods, a DGA [6] name detector required the extraction of human-defined statistical attributes to be effective. Instead, machine learning techniques automatically extract the necessary features during the training process while relying solely on the domain name string to make the required predictions, categorizing the names between DGA and legit. This feature is handy as malware is no longer aware of the components used to train model detectors. As a result, they cannot modify DGAs to generate names that are not detected based on these characteristics. Models trained in machine learning techniques are highly accurate and efficiently generalized, as presented in the relevant literature [7, 8].

The rest of the work includes Section 2, which provides an overview of the methods found in the literature and related to similar methods.  Section 3 describes in detail the motivation of this work.  Section 4 includes the methodology of the proposed system.  Section 4 explains the dataset used, and Section 5 analyzes the experiments for implementing the proposed approach. Finally, Section 6 summarizes the research conducted and presents the future objectives that can extend it.

## 2. Literature Review

With the increasing use of modern technologies in every field globally, the need to enhance the cyber security posture of educational organizations has significantly grown in recent years, especially in 2020 and after the COVID-19 pandemic. Many modern institutions have shifted their services to a remote-based approach. The research community has also focused on the education industry and distance learning to find innovative and privacy-preserving solutions to this new reality [2, 9].

Zhang et al. [10] reviewed current research on privacy-preserving technologies and deep collaborative learning. They concluded that each privacy-preserving technology has its own unique features. They asserted that while safe multiparty computing and homomorphic encryption can provide a high level of privacy and accuracy, the cost to users is a substantial computational and communication burden. Differential privacy, in which users input random noise into their data before sending it to the server, is a more practical and efficient solution. Nonetheless, it diminishes the model's accuracy. When huge businesses with sensitive data operate as users, homomorphic encryption technology is essential to assure the model's security. Differential privacy technology is required when a large number of people with little computer power operate as consumers to ensure the model's efficiency. A mix of safe multiparty computing, homomorphic encryption, and differential privacy is being used in a growing number of studies to provide a suitable trade-off between data privacy and utility.

Ryffel et al. [11] developed and explored a federated learning framework built on PyTorch for privacy-preserving deep learning. Their framework prioritized data ownership and secure processing. It proposed a representation based on command chains and tensors, allowing them to perform complicated privacy-preserving structures like federated learning, SMPC, and differential privacy while presenting a familiar deep learning API to the end-user. The Boston Housing and Pima Indian Diabetes datasets were used to test their implementation. Nonetheless, they discovered early in the development process that the framework added an enormous performance burden.

Bo Chang [12] used vignettes to depict various privacy scenarios in the online learning environment while researching student privacy issues in online learning environments. Because of the sharing of individual grades among group members and providing public input on blogs, his studies revealed direct legal ramifications of concern to students that are not entirely addressed in FERPA policy. Other privacy issues, such as open access to each other's work, transparent reflections, public comments, critical examination of assignments, and collaborative evaluations of students' work, arose in more nuanced ways. He suggested focusing on students' products rather than their names, informing students of the benefits they will receive and the contributions they will make by sharing their work publicly, and providing options for students to keep their identities private if they are uncomfortable about sharing their work publicly. To alleviate students' discomfort, he also emphasized that professors should educate students about the FERPA policy while adapting to partial privacy.

In his research study, Karunakaran [3] employed both public and real-time environmental datasets to detect text features and knowledge-based feature extraction to detect DGAs that randomly produced malicious domains. Because attackers only know how the DGA method works, he surveyed an algorithm to identify the DGA more efficiently. His model produced extremely excellent classification accuracy results. Finally, he suggested that by training and evaluating the dataset, he might improve the proposed technique.

From the above literature, we realize that researchers put their efforts into employing deep learning techniques and finding the best possible trade-off between privacy and utility because of the high processing power that these methods require.

## 3. Motivation

As it turned out, there are quite a few capable systems for categorizing DGAs, but their ability depends mainly on the dataset used for their training. Most of the published research uses DGA names that are publicly available and have resulted from published related projects and successful reverse-engineering efforts for various DGA families. The problem with these datasets is the limited number of names they consist of and, secondly, the large percentage of them that are obsolete [13]. Admittedly, they lack names from more recent DGAs, reducing the discernment of trainee models regarding emerging DGAs. On the other hand, organizations specializing in cyber security and ISP providers train their models, using for the training process data that they are in no way willing to share due to competition and financial interests. The problem becomes more realistic if we consider that one of the largest sources of both DGA or legit names, a recursive DNS server, obeys strict privacy protocols, making it impossible to access essential data (e.g., logs with DNS queries) for the best and most up-to-date training of a machine learning model [14, 15]. So, we come up with a scenario in which, while all stakeholders want to enhance their models, using the broadest possible variety and the most recent data available for training. No one wants to contribute to this direction by exposing their data to the public.

The above situation seems to lead to a dead end. To avoid compromising privacy while promoting scientific research into large datasets to improve digital security, it is imperative to simultaneously implement technical solutions to address data protection and usage requirements [16, 17]. A serious answer to this situation is using secured multiparty computation (SMPC) [18] cryptography to train a deep learning model, combining all available data during training without disclosure and meeting the requirements for privacy [7, 13, 19].

SMPC is a cryptographic technique that allows different parties to perform calculations through inputs while keeping these inputs private [18, 20, 21]. Essentially, in this model, a set of parts with private inputs performs distributed functions, ensuring the required privacy and security issues. Conceptually, SMPC replaces a reliable intermediary to implement reliable calculations [21–25].

## 4. Proposed Methodology

This work proposes a privacy-preserved Siamese variational-autoencoder for DGA network traffic identification. Variational autoencoders (VAEs) are neural networks that try to discover the internal structure of input data to produce similar data [26, 27]. In other words, these are models that try to display the characteristics of the data *φ*(*x*) and the categories *π*(*y*) in an embedding space. The main idea is that data from the same type should be displayed in the same area. The category description will be displayed, while data from different categories should be displayed in other areas [28, 29]. This creates a partition of the embedding space in |*Y*| areas. Then a simple architecture, such as a simple classifier, is used to learn this separation. It is trained to classify the common integration space points in |*Y*| possible classes. Finally, at the model evaluation time, the data (either belonging to known or unknown classes) is encoded in the embedding space and then classified into one of the available categories. The success of this approach is that the projection in the field of standard integration is made both from the areas of the known and unknown categories available during the training process [30, 31].

The proposed work uses Siamese, i.e., the parallel use of 2 autoencoders that encode and decode DGAs and their descriptions in one embedding space. To synchronize the areas of embedding space, the maximum mean discrepancy (MMD) metric is used in the model error function. Minimizing this amount synchronizes the probability distributions of DGAs and their categories in embedding space.

(1) $$\begin{array}{c} {ℒ}_{VAE}={ℒ}_{VAE_{1}}+{ℒ}_{VAE_{2}}=\beta D_{KL}\left(p_{E_{1}}\left(z|\phi \left(x\right)\right)\left\|\;\right.p\left(z\right)\right)-E_{p_{E_{1}}\left(z|\phi \left(x\right)\right)}\left[\log\;p_{D_{1}}\left(\phi \left(x\right)|z\right)\right]+\\ \beta D_{KL}\left(p_{E_{2}}\left(z|\pi \left(y\right)\right)\left\|\;\right.p\left(z\right)\right)-E_{p_{E_{2}}\left(z|\pi \left(y\right)\right)}\left[\log\;p_{D_{2}}\left(\pi \left(y\right)|z\right)\right]. \end{array}$$

where *ß* is the model hyperparameter, *E*_1_ and *D*_1_ are the encoder and decoder of DGA, and *E*_2_ and *D*_2_ are the encoder and decoder of the descriptions.

Siamese VAE encodes the characteristics *φ*(*x*) in a probabilistic profile, which is modeled as standard, so it depends on an average value and a scatter table. Then a point of the standard integration space *z N* (*μ*, Σ) is sampled and decoded. The error is added to the total error function of the model so that [27, 32]

The MMD ensures that the DGAs and descriptions are adequately decoded and form areas in the embedding space, but their distributions are synchronized by minimizing the Wasserstein distance to which it applies as follows [29, 33, 34]:

(2) $$\begin{array}{c} W_{p}\left(\mu ,\nu \right)≔{\left(\underset{\gamma \in \Gamma \left(\mu ,\nu \right)}{inf}\underset{M\times M}{\int }\text{d}x,y^{p}\text{d}\gamma \left(x,y\right)\right)}^{1/p}. \end{array}$$

Finally, the synchronization of the distributions is calculated as follows:

(3) $$\begin{array}{c} {ℒ}_{DA}=\sqrt{\mu_{1}-\mu_{2}^{2}+\sum_{1}^{1/2}-\sum_{2}^{1/2}2\text{ Frobenius}}. \end{array}$$

Although at this point the model works satisfactorily, the cross-synchronization technique is additionally used where

(4) $$\begin{array}{c} {ℒ}_{CA}=\phi \left(x\right)-D_{1}\left(E_{2}\left(\pi \left(y\right)\right)\right)+\pi \left(y\right)-D_{2}\left(E_{1}\left(\phi \left(x\right)\right)\right). \end{array}$$

The VAE of the descriptions is required to decode DGAs, and the DGA decoder to decode descriptions, so the total error function of the model is

(5) $$\begin{array}{c} ℒ\left(x,y;E,D\right)={ℒ}_{VAE}+\gamma {ℒ}_{CA}+\delta {ℒ}_{DA}. \end{array}$$

where *γ*, *δ* are hyperparameters of the model.

**Figure 1:**
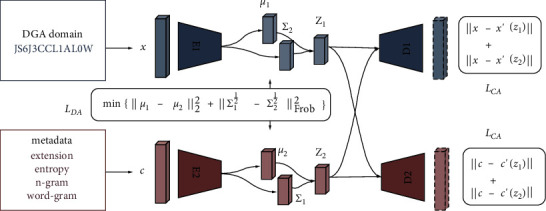
Siamese variational-autoencoder architecture.

Finally, a simple Softmax classifier is used to classify embedding space in |*Y*| categories. An indicative architecture of the proposed system is shown in Figure 1.

To engage stakeholders who wish to enhance their models using the broadest possible variety and the most recent data available for education without the potential exposure of their private data, a machine learning protocol based on the SMPC technique is implemented [20, 22]. The proposed function offers participants the same possibility, as it allows the calculation of its value *F* only through the exchange of messages between *n* participants. Such a calculation could theoretically be performed in the presence of an inviolable and trustworthy referee other than *n* the participants, to whom each would give his value *d*_*x*_, and he would correctly calculate its value *F* and announce to everyone only the result, as would be the case with the use of federated learning techniques.

In our case, we are interested in and present the implementation of a cumulative protocol for calculating the function (*d*_1_,…, *d*_*n*_) = *d*_1_ + *d*_2_ + ⋯ + *d*_*n*_ based on the Shamir secret sharing (3S) method for *n* participants with *n* threshold. The 3S algorithm is based on the secure splitting and sharing of information between several participants. Each of them receives a value unrelated to the secret (in this case, the training data of the machine learning model is considered a secret), called a share of the secret, which has no utility. The secret can only be recreated if several parts of it are combined. For a total number of n shares to be defined, the minimum number *t* ≤ *n* is set initially, called the threshold (*t*, *n*) required to recover the secret *S*. *t* − 1 random integers *a*_1_, *a*_2_,…, *a*_*k* − 1_ are selected while *a*_0_ = *S*, to implement the following polynomial [18, 22, 35]:

(6) $$\begin{array}{c} f\left(x\right)=a_{0}+a_{1}x+a_{2}x^{2}+\ldots +a_{t-1}x^{t-1}. \end{array}$$

Based on this polynomial, we obtain *n* random points (*i*, *f* (*i*))∶ ≠ 0. Each point is communicated to one of the *n* participants. Having the polynomial (*x*), for the value *x* = 0, we get the value (0) = *a*_0,_ which is the secret *S*. It is noted that to maintain the correct secrecy, all operations are done with elements of a finite field *F* with size *P* where *P* first number, greater than all the coefficient values of the polynomial and the values *t* and *n*.

For any subset of *t* points, the above polynomial can be reconstructed using the Lagrange interpolation. Specifically, let *n* + 1 points (*x*_0_, *y*_0_),…, (*x*_*j*_, *y*_*j*_),…, (*x*_*n*_, *y*_*n*_), where all *x*_*j*_ are different from each other. The Lagrange interpolation polynomial of *P*_*n*_ (*x*) degree ≤ *n* is given by the type as [22, 36]

(7) $$\begin{array}{c} P_{n\left(x\right)}=l_{0}\left(x\right)f\left(x_{0}\right)+l_{1}\left(x\right)f\left(x_{1}\right)+\ldots +l_{n}\left(x\right)f\left(x_{n}\right)=\sum_{i=0}^{n}l_{i}\left(x\right)f\left(x_{i}\right). \end{array}$$

with:

(8) $$\begin{array}{c} l_{i}\left(x\right)=\underset{0\leq j\leq n,j\neq i}{\prod }\frac{x-x_{j}}{x_{i}-x_{j}}. \end{array}$$

The remainder can be bound as

(9) $$\begin{array}{c} \left|Rx\right|\leq \frac{{\left(x_{k}-x_{0}\right)}^{k+1}}{\left(k+1\right)!}\underset{x_{0}\leq \xi \leq x_{k}}{\max}\left|f^{\left(k+1\right)}\left(\xi \right)\right|. \end{array}$$

The proposed protocol has the following steps [36, 37]:

1. Each participant *p* with a value *d* creates a random polynomial of *n*-th degree with a fixed value its hidden value *d*_*p*_, as *f*(*x*)=*d*_*p*_+*a*_1_*x*+*a*_2_*x*^2^+⋯+*a*_*n*_*x*^*n*^
2. Calculates *n* values of (*x*) for *n* different but predefined values *x*_*p*_ with *x*_*p*_ ≠ 0, one for each participant, including himself.
3. Sends to each participant *p* the corresponding value (*x*_*p*_).
4. Steps 1 to 3 are performed by all participants, and each one sends the corresponding values of the random polynomial (*x*). An essential element of the process is that *f*_*p*_ values are not sent randomly. Assuming that each default value *x*_*p*_ is assigned to a specific participant *p*, then the corresponding value of (*x*_*p*_) for the corresponding *x* must be sent to him by all participants.
5. Having each participant *p* receive *n* values, *f*_1_(*x*_*p*_), *f*_2_(*x*_*p*_),…, *f*_*n*_(*x*_*p*_) calculates their sum and notifies it to the other participants.
6. When all the sums have been announced, each participant uses them to perform Lagrange interpolation and reconstruct a new polynomial (*x*) equal to the sum of the random polynomials of all participants *f*_all_(*x*)=*f*_1_(*x*)+*f*_2_(*x*)+…+*f*_*n*_(*x*)
7. Given the sum of all polynomials, it is expected that the constant of *c*_*all*_ = *d*_1_ + *d*_2_ + ⋯ + *d*_*n*_ and is calculated for *x* = 0, (0) = c_all_*∑*.

## 5. Dataset

Two different sets of domain names were used to carry out the experiments. In the first dataset, 400,000 records are used from nonwordlist-based DGAs alone. Half of them came from Alexa's collection of the top 1 million randomly selected from the most popular names, and the rest were created by running specific DGA algorithms. Ten different DGAs were executed in more detail, and 20,000 names were generated for each of the above algorithms. The second dataset uses wordlist-based DGAs and includes 500,000 records, half of which came from the Alexa collection of the top 1 million randomly selected from the most popular names [4, 6]. The rest were created by executing ten different wordlist-based DGAs. It should be noted that we used domains that were related to education institutions (.ac,.edu, etc.), and e-learning software (Zoom, Cisco Webex, etc.)

**Figure 2:**
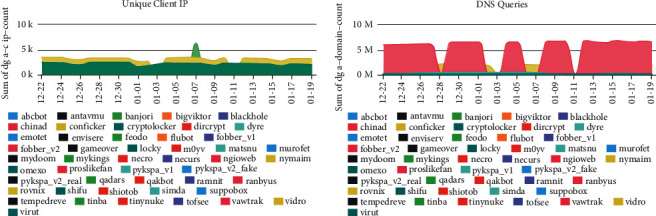
DGA domains unique client Ips and DNS queries by https://data.netlab.360.com/dga/.

An evaluation dataset consisting of 1,500,000 domain names was used to evaluate the method. Of these 800,000 legit domains, which are different from those of the training dataset, 550,000 come from the Alexa top 1 million collections, while domain name registration machines retrieved the rest. Respectively, of the 700,000 DGAs domains, 300,000 are real DGAs domains registered in corresponding lists of security organizations such as BlackHoleDNS, while the remaining 400,000 have been created after executing 40 different DGAs. The following Figure 2 illustrates the dataset.

The 20 algorithms have not been included in the training dataset to make the dataset more realistic. However, even for those included, different seeds and wordlists were used in their execution, so that the domain names generated were different from those used for training. For the creation of DGA domains, the length of the domains was random, ranging from 6 to 21 alphanumeric characters written in the Latin alphabet. An entropy algorithm was also applied to the creation of the domains as a degree of uncertainty to enhance the realism of the generated domains. Even with the Alexa grams technique, the degree of sequence between the generated DGA domain and the list of domains derived from Alexa was calculated using the technique of the probabilistic model for the prediction of the next n-gram element. Finally, the word-gram process was used to calculate the degree of correlation - sequence between the DGA domain and 500,000 widely used words to predict the next word-gram element.

The metadata used for the second VAE was the end of the domain name, the degree of entropy of each domain (entropy), the degree of the sequence of each domain (n-gram), and the degree of correlation-sequence of each domain (word-gram) [32, 34].

Overall, Table 1 presents the datasets used in this study as follows.

## 6. Experiments

To evaluate the performance of the proposed system per class as well as the estimation of the actual error during the training, we used the following measures [26, 38]:

Sensitivity=*tp*/pos, Specificity=*tn*/negat, Precision=*tp*/*tp*+*fp*, recall=*tp*/*tp*+*fn*, and *f* − score=2xpre x rec/pre+recaccuracy=sensitivity*∗*pos/pos +negat+specifivity*∗*neg/pos+ negat=*tp*+*tn*/pos+ negat

Where tn = true negative, tp = true positive, fn = false negative, and fp = false positive.

We conducted three experiments where, for the first time, training was performed with the nonwordlist-based DGA dataset and a test with the MixTest nonwordlist and wordlist DGA. Then training was done with the wordlist-based DGA dataset and testing was done with the MixTest nonwordlist and wordlist DGA. Finally, the two training datasets (nonwordlist-based DGA and wordlist-based DGA dataset) were combined, and the MixTrain nonwordlist was created and the wordlist DGA, which was tested with the MixTest nonwordlist and wordlist DGA. The following Table 2 shows the results of the followed procedure.

As can be seen from the table above, the generalizability of the system is significantly enhanced by the MixTrain (nonwordlist and wordlist-based DGA) dataset, which includes many more and much more complete samples of DGA domains.

In the case of applying the SMPC algorithm, we proved its functionality by proving that ring uniformities “retain” operations. Specifically, we demonstrated that there exists an isomorphism of rings of polynomials (i.e., be 1–1) where at least *R* is a transposition ring, and I is an ideal of *R* with [18, 25, 35]

(10) $$\begin{array}{c} \frac{R\left[t\right]}{I\left[t\right]}\overset{≅}{⟶}\left(\frac{R}{I}\right)\left[t\right]. \end{array}$$

In detail, considering the illustration as follows:

(11) $$\begin{array}{c} \Phi :\frac{R\left[t\right]}{I\left[t\right]}⟶\left(\frac{R}{I}\right)\left[t\right].,P\left(t\right)=\sum_{k=0}^{n}a_{k}t^{k}⟶\Phi \left(P\left(t\right)\right)=\sum_{k=0}^{n}\left(a_{k}+I\right)t^{k}. \end{array}$$

Then Φ(1_R[t]_) = Φ(1) = 1+ I = 1_(R/I)[t]_, i.e., the illustration Φ sends the unit of R[t] to its unit (R/I)[t].

If *P*(*t*)=∑_*k*=0_^*n*^*a*_*k*_*t*^*k*^ and *Q*(*t*)=∑_*k*=0_^*m*^*b*_*k*_*t*^*k*^ are two polynomials in the ring *R*[*t*], then we can assume without harm to the generality that *n* ≤ *m* and then we can write *P*(*t*)=∑_*k*=0_^*m*^*a*_*k*_*t*^*k*^, where we set *a*_n+1_ = ··· = *a*_m_ = 0. So, we will have

(12) $$\begin{array}{c} \Phi \left(P\left(t\right)+Q\left(t\right)\right)=\Phi \left(\sum_{k=0}^{m}a_{k}t^{k}+\sum_{k=0}^{m}b_{k}t^{k}\right)=\Phi \left(\sum_{k=0}^{m}\left(a_{k}+b_{k}\right)t^{k}\right)=\sum_{k=0}^{m}\left(a_{k}+b_{k}+I\right)t^{k}===\sum_{k=0}^{m}\left(a_{k}+I\right)t^{k}+\sum_{k=0}^{n}\left(b_{k}+I\right)t^{k}=\Phi \left(P\left(t\right)\right)+\Phi \left(Q\left(t\right)\right). \end{array}$$

Similarly, setting *c*_*k*_=∑_*l*=0_^*k*^*a*_*l*_*b*_*k*−*l*_, 0 ≤ *k* ≤ *n*+*m* we will have

(13) $$\begin{array}{c} \left.\Phi \left(P\left(t\right)\cdot Q\left(t\right)\right)=\Phi \left(\sum_{k=0}^{m}a_{k}t^{k}\cdot \sum_{k=0}^{m}b_{k}t^{k}\right)=\Phi \left(\sum_{k=0}^{n+m}c_{k}t^{k}\right)=\sum_{k=0}^{n+m}\left(c_{k}+I\right)t^{k}\right)=\sum_{k=0}^{n+m}\left(\sum_{l=0}^{k}\left(a_{l}+I\right)\left(b_{k-l}+I\right)\right)t^{k}=\sum_{k=0}^{n}\left(a_{k}+I\right)t^{k}\cdot \sum_{k=0}^{m}\left(b_{k}+I\right)t^{k}=\Phi \left(P\left(t\right)\right)\cdot \Phi \left(Q\left(t\right)\right). \end{array}$$

Thus, the illustration Φ is a homomorphism of rings which in addition is a homomorphism because if *A*(*t*)=∑_*k*=0_^*n*^(*a*_*k*_+*I*)*t*^*k*^ is a typical ring element (*R*/*I*)[*t*], then setting *P*(*t*)=∑_*k*=0_^*n*^*a*_*k*_*t*^*k*^ ∈ *R*[*t*] we will have Φ(P(*t*)) = A(*t*).

Let *P*(*t*)=∑_*k*=0_^*n*^*a*_*k*_*t*^*k*^ ∈ Ker(Φ), then Φ(*P*(*t*))=∑_*k*=0_^*n*^(*a*_*k*_+*I*)*t*^*k*^=0_(*R*/*I*)[*t*]_=*I* is the zero polynomial in the ring (*R*/*I*)[*t*], i.e., *a*_k_ + *I* = *I*, and therefore *a*_k_ ∈ I, 0 ≤ *k* ≤ *n*. This means that the polynomial *P*(*t*) ∈ *I*[*t*]. Conversely, if *P*(*t*) ∈*I*[*t*], then *a*_k_ ∈ I, 0 ≤ *k* ≤ *n*, and then obviously [21, 23]

(14) $$\begin{array}{c} \Phi \left(P\left(t\right)\right)=\sum_{k=0}^{n}\left(a_{k}+I\right)t^{k}=\sum_{k=0}^{n}\left(0_{R/I}\right)t^{k}=0_{\left(R/I\right)\left[t\right]}. \end{array}$$

Therefore

(15) $$\begin{array}{c} Ker\left(\Phi \right)=I\left[t\right]. \end{array}$$

and so, the subset *I*[*t*] is an ideal of *R*[*t*] as the nucleus of a ring homomorphism. Finally, because the imaging Φ is training, it follows that Φ induces the ring isomorphism of the original hypothesis as

(16) $$\begin{array}{c} \frac{R\left[t\right]}{I\left[t\right]}\overset{≅}{⟶}\left(\frac{R}{I}\right)\left[t\right]. \end{array}$$

We can therefore use the methodology of the SMPC algorithm to apply machine learning techniques with very high performance even in cases of encrypted data.

## 7. Discussion and Conclusions

The detection and timely assessment of DGA domains and DGA network traffic allows for the detection of incidents and the corresponding identification of correlations and relationships with security incidents, significantly mitigating the effects of sophisticated cyber-attacks. Individual efforts by independent actors cannot perform effectively and quickly in the field of knowledge discovery. On the contrary, collaborative efforts, which, as it turns out, can be implemented with remarkable learning models that also work on encrypted data, can lead to a significant increase in the accuracy of results and the generalization of learning models. Also, the increasing nature of the data requires the rise of training datasets, always considering the adaptation of the method to the available memory resources and computing power.

Considering the need for realistic and accurate security incident detection systems, this paper presented an innovative and highly practical privacy-preserved machine learning methodology for the timely detection of DGA domains and the network traffic they generate, with respect to distance education functionality. This methodology combines the Siamese variational-autoencoder in a complete framework. It is a robust system that calculates the number of maximum probable intervals within which an event is likely to occur based on a parametric evaluation that uses realistic datasets.

An essential advantage of the method, which has been proven experimentally, is that VAEs can, by receiving a combination of data and metadata, detect complex and sophisticated DGA domains. The dynamic identification of the proposed system directly integrates all the information in the sequence of the sample set, creating conditions for a realistic approach in recognizing security events.

Significant improvements in the evolution of the proposed system mainly concern the optimization of VAE hyperparameters, which are sensitive to modifications in determining the input data trend. Also, a significant improvement involves how the system is investigated with dynamic variational inference methodologies to provide a detailed approach to the subsequent probability of unobserved variables and apply a statistical conclusion for these variables.[39].

## Figures and Tables

**Table 1:** Training and test datasets.

Training datasets		Test dataset
Nonwordlist-based DGA	Wordlist-based DGA	MixTest nonwordlist and wordlist DGA
200.000 legit	250.000 legit	800.000 legit
200.000 DGA	250.000 DGA	700.000 DGA

**Table 2:** Results with various training datasets.

Training dataset	Accuracy	Recall	Precision	f-score
Nonwordlist-based DGA	0.8949	0.8883	0.8904	0.8903
Wordlist-based DGA	0.9072	0.9038	0.9056	0.9058
MixTrain (nonwordlist & wordlist-based DGA)	0.9260	0.9263	0.9259	0.9261

## Data Availability

The data used in this study are available from the author upon request.
